# Supplementary material for: Changes in the Aroma Profile and Phenolic Compound Contents of Different Strawberry Cultivars during Ripening
Source: Plants (Basel). 2024 May 20;13(10):1419. doi: 10.3390/plants13101419 (PMC11125040; doi:10.3390/plants13101419)

Supplementary Material: S1 Strawberry cultivars at five ripening stages of the fruit

Figure S1-1 Five ripening stages of the fruit of the cultivar 'Asia' (green fruit - R1, white fruit - R2, ripe red fruit - R3, fully ripe dark red fruit - R4 and overripe fruit - R5)

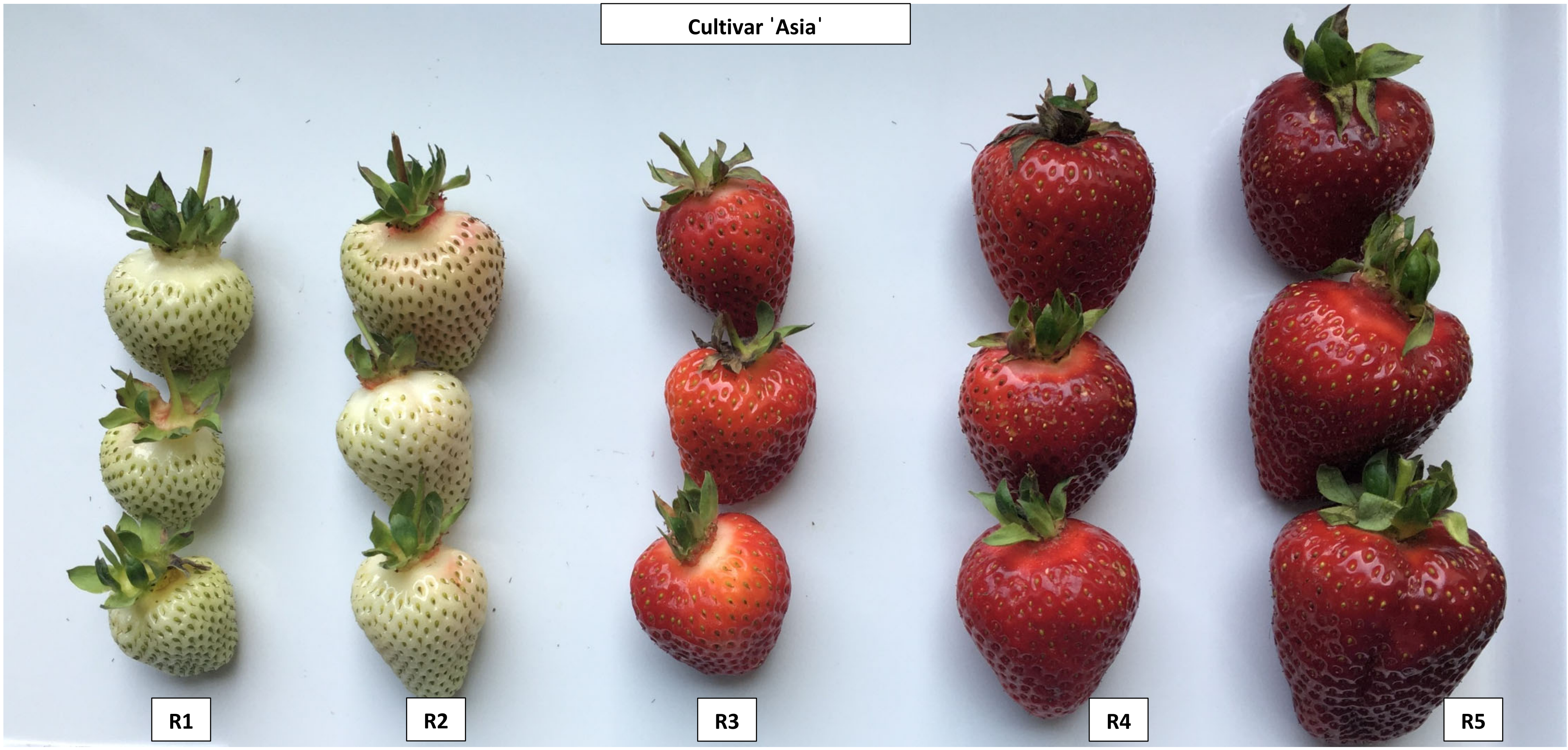

Figure S1-2 Five ripening stages of the fruit of the cultivar 'CIVN766' (green fruit - R1, white fruit - R2, ripe red fruit - R3, fully ripe dark red fruit - R4 and overripe fruit - R5)

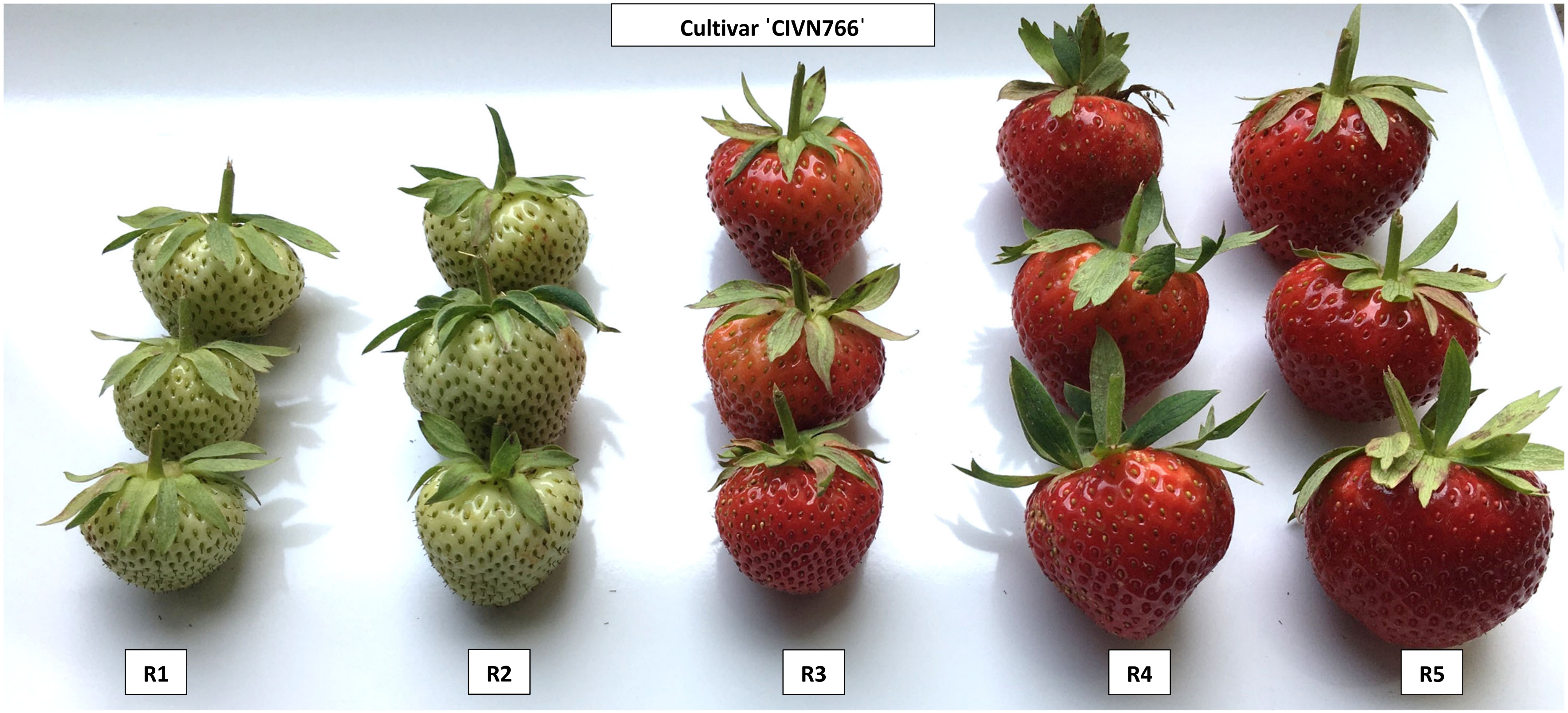

Figure S1-3 Five ripening stages of the fruit of the cultivar 'Clery' (green fruit - R1, white fruit - R2, ripe red fruit - R3, fully ripe dark red fruit - R4 and overripe fruit - R5)

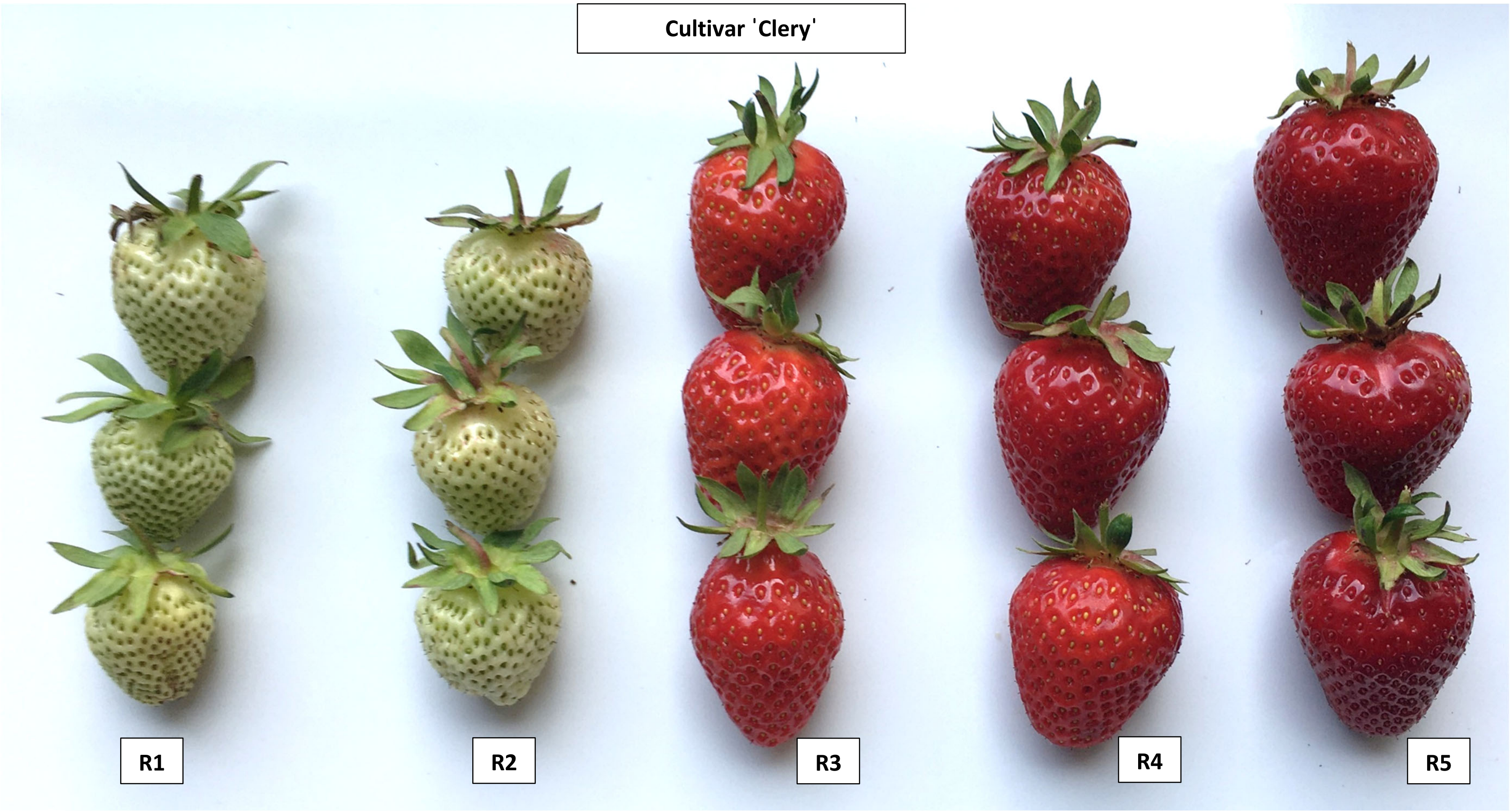

Figure S1-3 Five ripening stages of the fruit of the cultivar 'Malwina' (green fruit - R1, white fruit - R2, ripe red fruit - R3, fully ripe dark red fruit - R4 and overripe fruit - R5)

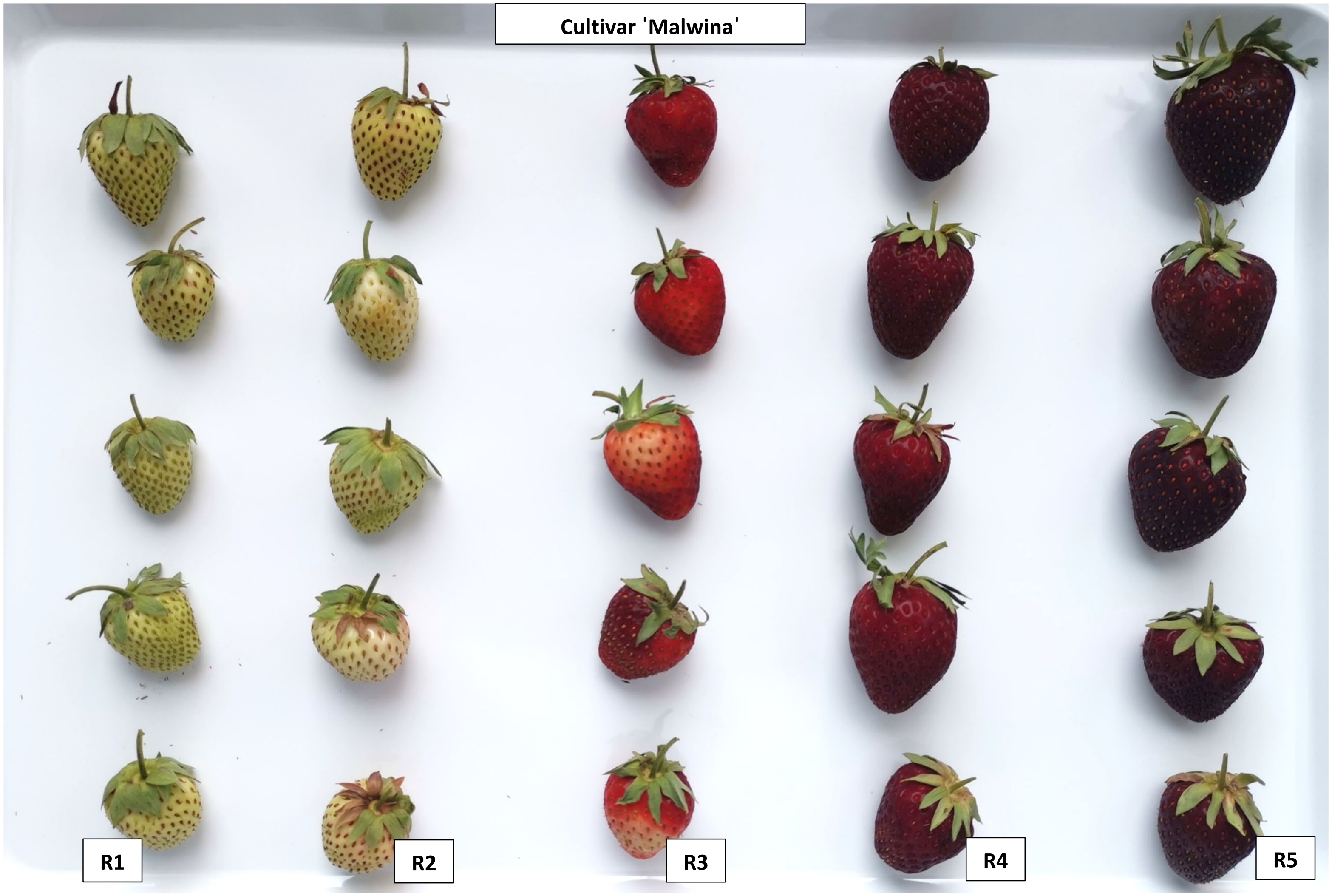

Supplement: Supplementary file 1 [file plants-13-01419-s001.zip › Supplementary Material S1 Ripening stages.pdf]
