# Supplementary material for: Changes in the Aroma Profile and Phenolic Compound Contents of Different Strawberry Cultivars during Ripening
Source: Plants (Basel). 2024 May 20;13(10):1419. doi: 10.3390/plants13101419 (PMC11125040; doi:10.3390/plants13101419)
Supplement: Supplementary file 1 [file plants-13-01419-s001.zip › Supplementary Material S2 MS phenolics.pdf]

Supplementary Material: S2 Identification of anthocyanins and other phenolic compounds in the 5 studied strawberry cultivars at 5 different ripening stages and the standards they are expressed as

Table S2-1 Identification of anthocyanins and other phenolic compounds in the 'Aprica' strawberry cultivar at 5 different ripening stages and the standards they are expressed as

	Positive mode $[M]^+$												
Peak No	Anthocyanin	λ [nm]	$[M+H]^+$ (*m/z*)	MS^2 (*m/z*)			Expressed as	Group	green (R1)	white (R2)	ripe (R3)	fully ripe (R4)	overripe (R5)
1A	cyanidin-3-*O*-β-galactoside	530	449	287			cyanidin-3-*O*-galactoside	Anthocyanins			x	x	x
2A	cyanidin 3-*O*-β-glucoside	530	449	287			cyanidin-3-*O*-glucoside	Anthocyanins			x	x	x
3A	pelargonidin 3-*O*-β-glucoside	530	433	271			pelargonidin-3-*O*-glucoside	Anthocyanins		x	x	x	x
4A	pelargonidin 3-*O*-β-rutinoside	530	579	271, 433			pelargonidin-3-*O*-glucoside	Anthocyanins			x	x	x
5A	cyanidin-3-(6″malonyl)glucoside	530	535	287			cyanidin-3-*O*-glucoside	Anthocyanins			x	x	x
6A	pelargonidin der.	530	519	271			pelargonidin-3-*O*-glucoside	Anthocyanins			x	x	x
7A	pelargonidin-3-(6″malonyl)glucoside	530	519	271, 475, 433			pelargonidin-3-*O*-glucoside	Anthocyanins			x	x	x
	Negative mode $[M-H]^-$												
Peak No	Phenolic compound	λ [nm]	$[M-H]^-$ (*m/z*)	MS^2 (*m/z*)	MS^3 (*m/z*)	MS^4 (*m/z*)	Expressed as	Group					
1	procyanidin dimer	280	577	425, 407, 451, 289			procyanidin B1	Flavanol	x	x	x	x	x
2	HHDP-galloylglucose	280	633	301, 494, 463, 226			ellagic acid	Hydroxybenzoic acid derivatives		x	x	x	x
3	procyanidin trimer	280	865	577, 407, 405, 287			procyanidin B1	Flavanol	x	x	x	x	x
4	*p*-coumaric acid hexoside	280, 350	325	163,145, 119			*p*-coumaric acid	Hydroxycinnamic acid derivatives			x	x	x
5	*p*-coumaric acid hexoside der.	280, 350	371	325	163, 145, 119		*p*-coumaric acid	Hydroxycinnamic acid derivatives			x	x	x
6	catechin	280, 350	335	289			catechin	Flavanol	x	x			
7	procyanidin trimer	280	865	577, 407, 405, 287			procyanidin B1	Flavanol	x				
8	apigenin-*O*-glucoside	280, 350	431	269, 217, 199			apigenin-7-glucoside	Flavonols	x	x			
9	apigenin-*O*-rhamnoside	280, 350	461	415	269,161		apigenin-7-glucoside	Flavonols			x	x	x
10	propelargonidin dimer	280, 350	561	289, 435, 544			procyanidin B1	Flavanol		x			
11	ferulic acid hexoside	280, 350	355	193, 217, 175			ferulic acid	Hydroxycinnamic acid derivatives		x	x	x	x
12	procyanidin tetramer	280	1153	865, 577, 407, 405, 287			procyanidin B1	Flavanol	x	x			
13	procyanidin trimer	280	865	577, 407, 405, 287			procyanidin B1	Flavanol	x	x			
14	propelargonidin dimer	280, 350	561	289, 435, 544			procyanidin B1	Flavanol		x	x	x	x
15	trigalloyl glucose	280	635	465	313, 447, 169		ellagic acid	Hydroxybenzoic acid derivatives	x				
16	caffeic acid der.	280	438	306, 288	254, 272, 287, 179	179,135	caffeic acid	Hydroxycinnamic acid derivatives			x	x	x
17	ferulic acid hexoside der.	280, 350	449	355, 269, 193			ferulic acid	Hydroxycinnamic acid derivatives	x	x	x		x
18	procyanidin dimer	280	577	425, 407, 451, 289			procyanidin B1	Flavanol	x	x	x		
19	procyanidin trimer	280	865	577, 407, 405, 287			procyanidin B1	Flavanol	x	x	x	x	x
20	ellagic acid der.	280, 350	463	301, 300, 257			ellagic acid	Hydroxybenzoic acid derivatives	x				
21	ellagic acid der.	280,350	465	301, 300			ellagic acid	Hydroxybenzoic acid derivatives	x	x			
22	procyanidin dimer	280	577	425, 407, 451, 289			procyanidin B1	Flavanol	x	x			
23	galloyl-bisHHDP-glucose	280	935	391, 301, 633			ellagic acid	Hydroxybenzoic acid derivatives	x	x			
24	*p*-coumaric acid hexoside	280, 350	325	163,145, 119			*p*-coumaric acid	Hydroxycinnamic acid derivatives	x	x	x	x	x
25	galloyl-bisHHDP-glucose	280	935	391, 301, 633			ellagic acid	Hydroxybenzoic acid derivatives	x	x			
26	ellagic acid-O-deoxyhexoside	280, 350	447	301, 300	257, 229		ellagic acid	Hydroxybenzoic acid derivatives	x	x	x	x	x
27	ellagic acid-O-deoxyhexoside	280, 350	447	301, 300	257, 229		ellagic acid	Hydroxybenzoic acid derivatives	x	x	x	x	x
28	rutin	350	609	301, 300			kaempferol-3-glucoside	Flavonols			x	x	x
29	galloyl-bisHHDP-glucose	280	935	391, 301, 633			ellagic acid	Hydroxybenzoic acid derivatives	x				
30	cinnamic acid-3-*O*-hexoside	280, 350	355	309, 147, 207, 248			caffeic acid	Hydroxycinnamic acid derivatives	x	x	x	x	x
31	isoquercetin	350	463	301, 300	179, 151		quercetin-3-glucoside	Flavonols	x	x			
32	kaempferol-3-coumaroylhexoside	350	593	447, 285, 257, 229			kaempferol-3-glucoside	Flavonols			x	x	x
33	caffeic acid der.	280	403	358, 305, 385			caffeic acid	Hydroxycinnamic acid derivatives			x	x	x
34	quercetin-3-*O*-β-glucuronide	280, 350	477	301	179, 151		quercetin-3-glucoside	Flavonols	x	x	x	x	x
35	isorhamnetin-3-*O*-glucoside	350	477	433, 301, 179			isorhamnetin-3-glucoside	Flavonols		x	x	x	x
36	kaempferol-*O*-hexoside	350	447	284, 285			kaempferol-3-glucoside	Flavonols	x	x	x	x	x
37	*p*-coumaric acid hexoside	280, 350	325	163,145, 119			p-coumaric acid	Hydroxycinnamic acid derivatives			x	x	x
38	kaempferol-3-*O*-β-glucuronide	280, 350	461	285	257, 267, 241		kaempferol-3-glucoside	Flavonols	x	x	x	x	x
39	isorhamnetin-3-*O*-β-glucuronide	350	491	315	300		isorhamnetin-3-glucoside	Flavonols	x	x	x	x	x
40	kaempferol acetyl hexoside	350	489	285	257, 267, 241		kaempferol-3-glucoside	Flavonols		x	x	x	x

der., derivative; $[M^-H]^-$, pseudo-molecular ion identified in negative ion mode; M^+, pseudo-molecular ion identified in positive ion mode ion.

Table S2-2 Identification of anthocyanins and other phenolic compounds in the 'Asia' strawberry cultivar at 5 different ripening stages and the standards they are expressed as

	Positive mode [M]$^+$												
Peak No	Anthocyanin	λ [nm]	[M+ H]$^+$ (*m/z*)	MS^2 (*m/z*)			Expressed as	Group	green (R1)	white (R2)	ripe (R3)	fully ripe (R4)	overripe (R5)
1A	cyanidin-3-*O*-β-galactoside	530	449	287			cyanidin-3-*O*-galactoside	Anthocyanins			x	x	x
2A	cyanidin 3-*O*-β-glucoside	530	449	287			cyanidin-3-*O*-glucoside	Anthocyanins			x	x	x
3A	pelargonidin 3-*O*-β-glucoside	530	433	271			pelargonidin-3-*O*-glucoside	Anthocyanins		x	x	x	x
4A	pelargonidin 3-*O*-β-rutinoside	530	579	271, 433			pelargonidin-3-*O*-glucoside	Anthocyanins			x	x	x
5A	pelargonidin-3-(6″malonyl)glucoside	530	519	271, 475, 433			pelargonidin-3-*O*-glucoside	Anthocyanins			x	x	x
6A	pelargonidin-3-*O*-acetylglucoside	530	475	271			pelargonidin-3-*O*-glucoside	Anthocyanins			x	x	x
	Negative mode [M- H]$^-$												
Peak No	Phenolic compound	λ [nm]	[M- H]$^-$ (*m/z*)	MS^2 (*m/z*)	MS^3 (*m/z*)	MS^4 (*m/z*)	Expressed as	Group					
1	procyanidin dimer	280	577	425, 407, 451, 289			procyanidin B1	Flavanol	x	x	x	x	x
2	HHDP-galloylglucose	280	633	301, 494, 463, 226			ellagic acid	Hydroxybenzoic acid derivatives		x	x	x	x
3	procyanidin trimer	280	865	577, 407, 405, 287			procyanidin B1	Flavanol	x	x	x	x	x
4	*p*-coumaric hexoside	280, 350	325	163,145, 119			*p*-coumaric acid	Hydroxycinnamic acid derivatives			x	x	x
5	catechin	280, 350	335	289			catechin	Flavanol	x	x			
6	p-coumaric hexoside der.	280, 350	361	325	163, 145, 119		*p*-coumaric acid	Hydroxycinnamic acid derivatives			x	x	x
7	apigenin-*O*-glucoside	280, 350	431	269, 217, 199			apigenin-7-glucoside	Flavonols	x	x			
8	apigenin-*O*-rhamnoside	350, 350	461	415	269,161		apigenin-7-glucoside	Flavonols	x	x	x	x	x
9	ferulic acid hexoside	280, 350	355	193, 217, 175			ferulic acid	Hydroxycinnamic acid derivatives	x	x	x	x	x
10	*p*-coumaric acid hexoside	280, 350	325	163,145, 119			*p*-coumaric acid	Hydroxycinnamic acid derivatives		x			
11	propelargonidin dimer	280, 350	561	289, 435, 544			procyanidin B1	Flavanol	x	x	x	x	x
12	trigalloyl glucose	280	635	465	313, 447, 169		ellagic acid	Hydroxybenzoic acid derivatives	x				
13	ellagic acid	280	301	301, 283			ellagic acid	Hydroxybenzoic acid derivatives	x				
14	ferulic acid hexoside der.	280, 350	449	355, 269, 193			ferulic acid	Hydroxycinnamic acid derivatives	x	x	x	x	x
15	ellagic acid der.	280	463	301, 300, 257			ellagic acid	Hydroxybenzoic acid derivatives	x	x	x	x	x
16	apigenin-*O*-rhamnoside	350, 350	461	415	269,161		apigenin-7-glucoside	Flavonols	x				
17	procyanidin trimer	280	865	577, 407, 405, 287			procyanidin B1	Flavanol	x				
18	procyanidin dimer	280	577	425, 407, 451, 289			procyanidin B1	Flavanol	x	x			
19	galloyl-HHDP-DHHDP-hexose	280	951	933, 907, 781			ellagic acid	Hydroxybenzoic acid derivatives		x			
20	ellagic acid der.	280	579	447, 433, 403	323, 301, 300		ellagic acid	Hydroxybenzoic acid derivatives				x	x
21	galloyl-bisHHDP-glucose	280	935	391, 301, 633			ellagic acid	Hydroxybenzoic acid derivatives	x				
22	ellagic acid der.	280	579	447, 433, 403	323, 301, 300		ellagic acid	Hydroxybenzoic acid derivatives	x	x	x		
23	tetramethyl-ellagic acid-hexose	280	521	359, 341			ellagic acid	Hydroxybenzoic acid derivatives	x				
24	ellagic acid pentoside	280	433	301, 300, 313			ellagic acid	Hydroxybenzoic acid derivatives	x	x	x	x	x
25	ellagic acid-*O*-deoxyhexoside	280, 350	447	301, 300	257, 229		ellagic acid	Hydroxybenzoic acid derivatives	x	x	x	x	x
26	rutin	350	609	301, 300			kaempferol-3-glucoside	Flavonols	x	x	x	x	x
27	cinnamic acid-3-*O*-hexoside	280, 350	355	309, 147, 207, 248			caffeic acid	Hydroxycinnamic acid derivatives	x	x	x	x	x
28	kaempferol-3-coumaroylhexoside	350	593	447, 285, 257, 229			kaempferol-3-glucoside	Flavonols			x	x	x
29	caffeic acid der.	280	403	358, 305, 385			caffeic acid	Hydroxycinnamic acid derivatives	x	x	x	x	x
30	quercetin-3-*O*-β-glucuronide	280, 350	477	301	179, 151		quercetin-3-glucoside	Flavonols	x	x	x	x	x
31	kaempferol-*O*-hexoside	350	447	284, 285			kaempferol-3-glucoside	Flavonols	x	x	x	x	x
32	p-coumaric hexoside	280, 350	325	163,145, 119			p-coumaric acid	Hydroxycinnamic acid derivatives			x	x	x
33	ellagic acid der.	350	505	301, 300, 343, 445			ellagic acid	Hydroxybenzoic acid derivatives	x	x	x	x	x
34	kaempferol-3-*O*-β-glucuronide	280, 350	461	285	257, 267, 241		kaempferol-3-glucoside	Flavonols	x	x	x	x	x
35	isorhamnetin-3-*O*-β-glucuronide	350	491	315	300		isorhamnetin-3-glucoside	Flavonols	x	x	x	x	x
36	kaempferol acetyl hexoside	350	489	285	257, 267, 241		kaempferol-3-glucoside	Flavonols			x	x	x

der., derivative; [M$^-$H]$^-$, pseudo-molecular ion identified in negative ion mode; M$^+$, pseudo-molecular ion identified in positive ion mode ion.

Table S2-3 Identification of anthocyanins and other phenolic compounds in the 'CIVN766' strawberry cultivar at 5 different ripening stages and the standards they are expressed as

Positive mode [M]⁺													
Peak No	Anthocyanin	λ [nm]	[M+ H]⁺ (*m/z*)	MS² (*m/z*)			Expressed as	Group	green (R1)	white (R2)	ripe (R3)	fully ripe (R4)	overripe (R5)
1A	cyanidin-3-*O*-β-galactoside	530	449	287			cyanidin-3-*O*-galactoside	Anthocyanins	x	x	x	x	x
2A	cyanidin 3-*O*-β-glucoside	530	449	287			cyanidin-3-*O*-glucoside	Anthocyanins			x	x	x
3A	pelargonidin 3-*O*-β-glucoside	530	433	271			pelargonidin-3-*O*-glucoside	Anthocyanins	x	x	x	x	x
4A	cyanidin-3-(6″malonyl)glucoside	530	535	287			cyanidin-3-*O*-glucoside	Anthocyanins		x	x	x	x
5A	pelargonidin der.	530	519	271			pelargonidin-3-*O*-glucoside	Anthocyanins			x	x	x
6A	pelargonidin-3-(6″malonyl)glucoside	530	519	271, 475, 433			pelargonidin-3-*O*-glucoside	Anthocyanins			x	x	x
Negative mode [M- H]⁻													
Peak No	Phenolic compound	λ [nm]	[M- H]⁻ (*m/z*)	MS² (*m/z*)	MS³ (*m/z*)	MS⁴ (*m/z*)	Expressed as	Group					
1	procyanidin dimer	280	577	425, 407, 451, 289			procyanidin B1	Flavanol	x	x	x	x	x
2	HHDP-galloylglucose	280	633	301, 494, 463, 226			ellagic acid	Hydroxybenzoic acid derivatives	x	x	x	x	x
3	procyanidin trimer	280	865	577, 407, 405, 287			procyanidin B1	Flavanol	x	x	x	x	x
4	*p*-coumaric acid hexoside	280, 350	325	163,145, 119			*p*-coumaric acid	Hydroxycinnamic acid derivatives			x	x	x
5	*p*-coumaric acid hexoside der.	280, 350	371	325	163, 145, 119		*p*-coumaric acid	Hydroxycinnamic acid derivatives			x	x	x
6	catechin	280, 350	335	289			catechin	Flavanol	x	x	x	x	x
7	procyanidin tetramer	280	1153	865, 577, 407, 405, 287			procyanidin B1	Flavanol	x	x			
8	apigenin-O-glucoside	280, 350	431	269, 217, 199			apigenin-7-glucoside	Flavonols		x	x	x	x
9	apigenin-O-rhamnoside	350, 350	461	415	269,161		apigenin-7-glucoside	Flavonols	x	x			
10	propelargonidin dimer	280, 350	561	289, 435, 544			procyanidin B1	Flavanol	x	x	x	x	x
11	ferulic acid hexoside	280, 350	355	193, 217, 175			ferulic acid	Hydroxycinnamic acid derivatives	x	x	x	x	x
12	monogalloyl glucose	280	331	313, 169, 151, 125			procyanidin B1	Flavanol	x	x	x	x	x
13	procyanidin tetramer	280	1153	865, 577, 407, 405, 287			procyanidin B1	Flavanol	x				
14	propelargonidin trimer	280	865	561	289, 435, 544		procyanidin B1	Flavanol	x	x			
15	propelargonidin dimer	280, 350	561	289, 435, 544			procyanidin B1	Flavanol	x	x			
16	ferulic acid hexoside der.	280, 350	449	355, 269, 193			ferulic acid	Hydroxycinnamic acid derivatives	x	x	x	x	x
17	procyanidin dimer	280	577	425, 407, 451, 289			procyanidin B1	Flavanol	x	x	x	x	x
18	procyanidin dimer	280	577	425, 407, 451, 289			procyanidin B1	Flavanol	x	x			
19	ellagic acid pentoside	280, 350	433	301, 300, 313			ellagic acid	Hydroxybenzoic acid derivatives		x			
20	tetramethyl ellagic acid hexose	280, 350	521	359, 341			ellagic acid	Hydroxybenzoic acid derivatives			x	x	x
21	ellagic acid der.	280, 350	595	300, 301, 445, 463, 475			ellagic acid	Hydroxybenzoic acid derivatives		x			
22	*p*-coumaric acid hexoside	280, 350	325	163,145, 119			*p*-coumaric acid	Hydroxycinnamic acid derivatives	x	x			
23	galloyl-bisHHDP-glucose	280	935	391, 301, 633			ellagic acid	Hydroxybenzoic acid derivatives	x	x	x	x	x
24	ellagic acid-*O*-deoxyhexoside	280, 350	447	301, 300	257, 229		ellagic acid	Hydroxybenzoic acid derivatives	x	x	x	x	x
25	ellagic acid-*O*-deoxyhexoside	280, 350	447	301, 300	257, 229		ellagic acid	Hydroxybenzoic acid derivatives	x	x	x	x	x
26	ellagic acid	280	301	301, 283			ellagic acid	Hydroxybenzoic acid derivatives	x	x	x	x	x
27	cinnamic acid-3-*O*-hexoside	280, 350	355	309, 147, 207, 248			caffeic acid	Hydroxycinnamic acid derivatives	x	x	x	x	x
28	isoquercetin	350	463	301, 300	179, 151		quercetin-3-glucoside	Flavonols	x	x	x	x	x
29	caffeic acid der.	280	403	358, 305, 385			caffeic acid	Hydroxycinnamic acid derivatives		x	x	x	x
30	quercetin-3-*O*-β-glucuronide	280, 350	477	301	179, 151		quercetin-3-glucoside	Flavonols	x	x	x	x	x
31	isorhamnetin-3-*O*-glucoside	350	477	433, 301, 179			isorhamnetin-3-glucoside	Flavonols	x	x	x	x	x
32	kaempferol-*O*-hexoside	350	447	284, 285			kaempferol-3-glucoside	Flavonols	x	x	x	x	x
33	*p*-coumaric acid hexoside	280, 350	325	163,145, 119			*p*-coumaric acid	Hydroxycinnamic acid derivatives			x	x	x
34	kaempferol-3-*O*-β-glucuronide	280, 350	461	285	257, 267, 241		kaempferol-3-glucoside	Flavonols	x	x	x	x	x
35	caffeic acid der.	280	417	223, 179, 383	179, 135		caffeic acid	Hydroxycinnamic acid derivatives			x	x	x

der., derivative; $[M^-H]^-$, pseudo-molecular ion identified in negative ion mode; M^+, pseudo-molecular ion identified in positive ion mode ion.

Table S2-4 Identification of anthocyanins and other phenolic compounds in the 'Clery' strawberry cultivar at 5 different ripening stages and the standards they are expressed as

Positive mode [M]+													
Peak No	Anthocyanin	λ [nm]	[M+ H]+ (*m/z*)	MS2 (*m/z*)			Expressed as	Group	green (R1)	white (R2)	ripe (R3)	fully ripe (R4)	overripe (R5)
1A	cyanidin-3-*O*-β-galactoside	530	449	287			cyanidin-3-*O*-galactoside	Anthocyanins		x	x	x	x
2A	cyanidin 3-*O*-β-glucoside	530	449	287			cyanidin-3-*O*-glucoside	Anthocyanins			x	x	x
3A	pelargonidin 3-*O*-β-glucoside	530	433	271			pelargonidin-3-*O*-glucoside	Anthocyanins		x	x	x	x
4A	pelargonidin 3-*O*-β-rutinoside	530	579	271, 433			pelargonidin-3-*O*-glucoside	Anthocyanins			x	x	x
5A	cyanidin-3-(6″malonyl)glucoside	530	535	287			cyanidin-3-*O*-glucoside	Anthocyanins			x	x	x
6A	pelargonidin der.	530	519	271			pelargonidin-3-*O*-glucoside	Anthocyanins			x	x	x
7A	pelargonidin-3-(6″malonyl)glucoside	530	519	271, 475, 433			pelargonidin-3-*O*-glucoside	Anthocyanins			x	x	x
Negative mode [M- H]-													
Peak No	Phenolic compound	λ [nm]	[M- H]- (*m/z*)	MS2 (*m/z*)	MS3 (*m/z*)	MS4 (*m/z*)	Expressed as	Group					
1	procyanidin dimer	280	577	425, 407, 451, 289			procyanidin B1	Flavanol	x	x	x	x	x
2	HHDP-galloylglucose	280	633	301, 494, 463, 226			ellagic acid	Hydroxybenzoic acid derivatives	x				
3	catechin	280, 350	335	289			catechin	Flavanol	x	x			
4	*p*-coumaric acid hexoside	280, 350	325	163,145, 119			*p*-coumaric acid	Hydroxycinnamic acid derivatives			x	x	x
5	*p*-coumaric acid hexoside der.	280, 350	371	325	163, 145, 119		*p*-coumaric acid	Hydroxycinnamic acid derivatives			x	x	x
6	procyanidin trimer	280	865	577, 407, 405, 287			procyanidin B1	Flavanol	x				
7	apigenin-O-rhamnoside	280, 350	461	415	269,161		apigenin-7-glucoside	Flavonols	x	x			
8	ferulic acid hexoside	280, 350	355	193, 217, 175			ferulic acid	Hydroxycinnamic acid derivatives			x	x	x
9	ellagic acid-O-deoxyhexoside	280, 350	447	301, 300	257, 229		ellagic acid	Hydroxybenzoic acid derivatives				x	x
10	propelargonidin dimer	280, 350	561	289, 435, 544			procyanidin B1	Flavanol	x				
11	trigalloyl glucose	280	635	465	313, 447, 169		ellagic acid	Hydroxybenzoic acid derivatives		x			
12	caffeic acid der.	280, 350	581	563, 383, 401	223, 179, 365, 159	179, 135	caffeic acid	Hydroxycinnamic acid derivatives			x	x	x
13	procyanidin dimer	280	577	425, 407, 451, 289			procyanidin B1	Flavanol		x			
14	caffeic acid der.	280	438	306, 288	254, 272, 287, 179	179,135	caffeic acid	Hydroxycinnamic acid derivatives			x	x	x
15	apigenin-*O*-rhamnoside	280, 350	461	415	269,161		apigenin-7-glucoside	Flavonols	x		x	x	x
16	procyanidin trimer	280	865	577, 407, 405, 287			procyanidin B1	Flavanol		x			
17	ferulic acid hexoside der.	280, 350	449	355, 269, 193			ferulic acid	Hydroxycinnamic acid derivatives			x	x	x
18	procyanidin dimer	280	577	425, 407, 451, 289			procyanidin B1	Flavanol		x			
19	galloyl-bisHHDP-glucose	280	467	391, 301, 633			ellagic acid	Hydroxybenzoic acid derivatives	x				
20	*p*-coumaric acid hexoside	280	325	163, 119			*p*-coumaric acid	Hydroxycinnamic acid derivatives	x				
21	ellagic acid der.	280, 350	479	301, 300, 433	257, 229, 185		ellagic acid	Hydroxybenzoic acid derivatives			x	x	x
22	galloyl-bisHHDP-glucose	280	935	391, 301, 633			ellagic acid	Hydroxybenzoic acid derivatives		x			
23	galloyl-bisHHDP-glucose	280	467	391, 301, 633			ellagic acid	Hydroxybenzoic acid derivatives	x				
24	*p*-coumaric acid hexoside	280, 350	325	163,145, 119			*p*-coumaric acid	Hydroxycinnamic acid derivatives		x	x	x	x
25	galloyl-bisHHDP-glucose	280	935	391, 301, 633			ellagic acid	Hydroxybenzoic acid derivatives		x			
26	ellagic acid der.	350	595	301, 300, 445, 463, 475			ellagic acid	Hydroxybenzoic acid derivatives		x	x	x	
27	ellagic acid-*O*-deoxyhexoside	280, 350	447	301, 300	257, 229		ellagic acid	Hydroxybenzoic acid derivatives	x	x	x	x	x
28	ellagic acid-*O*-deoxyhexoside	350	447	301, 300	257, 229		ellagic acid	Hydroxybenzoic acid derivatives	x	x	x	x	x
29	rutin	350	609	301, 300			kaempferol-3-glucoside	Flavonols					
30	cinnamic acid-3-*O*-hexoside	280, 350	355	309, 147, 207, 248			caffeic acid	Hydroxycinnamic acid derivatives	x	x	x	x	x
31	isoquercetin	350	463	301, 300	179, 151		quercetin-3-glucoside	Flavonols	x	x	x	x	x
32	kaempferol-3-coumaroylhexoside	350	593	447, 285, 257, 229			kaempferol-3-glucoside	Flavonols			x	x	x
33	caffeic acid der.	280	403	358, 305, 385			caffeic acid	Hydroxycinnamic acid derivatives			x	x	x
34	quercetin-3-*O*-β-glucuronide	280, 350	477	301	179, 151		quercetin-3-glucoside	Flavonols	x	x	x	x	x
35	kaempferol-*O*-hexoside	350	447	284, 285			kaempferol-3-glucoside	Flavonols	x	x	x	x	x
36	kaempferol-3-*O*-β-glucuronide	280, 350	461	285	257, 267, 241		kaempferol-3-glucoside	Flavonols	x	x	x	x	x
37	isorhamnetin-3-*O*-β-glucuronide	350	491	315	300		isorhamnetin-3-glucoside	Flavonols	x	x	x	x	x

der., derivative; [M-H]-, pseudo-molecular ion identified in negative ion mode; M+, pseudo-molecular ion identified in positive ion mode ion.

Table S2-5 Identification of anthocyanins and other phenolic compounds in the 'Malwina' strawberry cultivar at 5 different ripening stages and the standards they are expressed as

	Positive mode [M]⁺												
Peak No	Anthocyanin	λ [nm]	[M+ H]⁺ (*m/z*)	MS^2 (*m/z*)			Expressed as	Group	green (R1)	white (R2)	ripe (R3)	fully ripe (R4)	overripe (R5)
1A	cyanidin-3-*O*-β-galactoside	530	449	287			cyanidin-3-*O*-galactoside	Anthocyanins	x	x	x	x	x
2A	cyanidin 3-*O*-β-glucoside	530	449	287			cyanidin-3-*O*-glucoside	Anthocyanins			x	x	x
3A	pelargonidin 3-*O*-β-glucoside	530	433	271			pelargonidin-3-*O*-glucoside	Anthocyanins	x	x	x	x	x
4A	pelargonidin 3-*O*-β-rutinoside	530	579	271, 433			pelargonidin-3-*O*-glucoside	Anthocyanins			x	x	x
5A	pelargonidin 3-*O*-β-arabinoside	530	403	271			pelargonidin-3-*O*-glucoside	Anthocyanins			x	x	x
6A	cyanidin-3-(6″malonyl)glucoside	530	535	287			cyanidin-3-*O*-glucoside	Anthocyanins		x	x	x	x
7A	pelargonidin der.	530	519	271			pelargonidin-3-*O*-glucoside	Anthocyanins			x	x	x
8A	pelargonidin-3-(6″malonyl)glucoside	530	519	271, 475, 433			pelargonidin-3-*O*-glucoside	Anthocyanins			x	x	x
9A	5-pyranopelargonidin-3-glucoside	530	501	339			pelargonidin-3-*O*-glucoside	Anthocyanins			x	x	x
10A	pelargonidin-3-*O*-acetylglucoside	530	475	271			pelargonidin-3-*O*-glucoside	Anthocyanins			x	x	x
	Negative mode [M- H]⁻												
Peak No	Phenolic compound	λ [nm]	[M- H]⁻ (*m/z*)	MS^2 (*m/z*)	MS^3 (*m/z*)	MS^4 (*m/z*)	Expressed as	Group					
1	procyanidin dimer	280	577	425, 407, 451, 289			procyanidin B1	Flavanol	x	x	x	x	x
2	HHDP-galloylglucose	280	633	301, 494, 463, 226			ellagic acid	Hydroxybenzoic acid derivatives	x				
3	procyanidin trimer	280	865	577, 407, 405, 287			procyanidin B1	Flavanol	x	x	x	x	x
4	*p*-coumaric acid hexoside	280, 350	325	163,145, 119			*p*-coumaric acid	Hydroxycinnamic acid derivatives			x	x	x
5	*p*-coumaric acid hexoside der.	280, 350	371	325	163, 145, 119		*p*-coumaric acid	Hydroxycinnamic acid derivatives		x	x	x	x
6	catechin	280, 350	335	289			catechin	Flavanol	x	x			
7	procyanidin tetramer	280	1153	865, 577, 407, 405, 287			procyanidin B1	Flavanol	x				
8	procyanidin dimer	280	577	425, 407, 451, 289			procyanidin B1	Flavanol	x				
9	apigenin pentose der.	280, 350	585	547	401, 269, 161		apigenin-7-glucoside	Flavonols		x			
10	apigenin-O-glucoside	280, 350	431	269, 217, 199			apigenin-7-glucoside	Flavonols			x	x	x
11	apigenin-O-rhamnoside	350, 350	461	415	269,161		apigenin-7-glucoside	Flavonols		x			
12	propelargonidin dimer	280, 350	561	289, 435, 544			procyanidin B1	Flavanol		x			
13	ferulic acid hexoside	280, 350	355	193, 217, 175			ferulic acid	Hydroxycinnamic acid derivatives		x	x	x	x
14	procyanidin tetramer	280	1153	865, 577, 407, 405, 287			procyanidin B1	Flavanol	x				
15	propelargonidin dimer	280, 350	561	289, 435, 544			procyanidin B1	Flavanol			x	x	x
16	brevifolin carboxylic acid	280, 350	291	247	219, 191, 203, 175	191	ellagic acid	Hydroxybenzoic acid derivatives	x	x			
17	ferulic acid hexoside der.	280, 350	449	355, 269, 193			ferulic acid	Hydroxycinnamic acid derivatives	x	x	x	x	x
18	ellagic acid der.	280,350	463	301, 300, 257			ellagic acid	Hydroxybenzoic acid derivatives	x	x	x	x	x
19	ellagic acid	280,350	301	301, 283			ellagic acid	Hydroxybenzoic acid derivatives	x				
20	*p*-coumaric acid hexoside	280, 350	325	163,145, 119			*p*-coumaric acid	Hydroxycinnamic acid derivatives	x				
21	galloyl-bisHHDP-glucose	280	935	391, 301, 633			ellagic acid	Hydroxybenzoic acid derivatives	x				
22	ellagic acid der.	280, 350	479	301, 300, 433	257, 229, 185		ellagic acid	Hydroxybenzoic acid derivatives				x	x
23	ellagic acid der.	280, 350	933	631, 479. 451, 301, 300			ellagic acid	Hydroxybenzoic acid derivatives		x			
24	ellagic acid pentoside	280, 350	433	301, 300, 313			ellagic acid	Hydroxybenzoic acid derivatives	x	x	x	x	x
25	ellagic acid-*O*-deoxyhexoside	280, 350	447	301, 300	257, 229		ellagic acid	Hydroxybenzoic acid derivatives	x	x	x	x	x
26	ellagic acid-*O*-deoxyhexoside	280, 350	447	301, 300	257, 229		ellagic acid	Hydroxybenzoic acid derivatives	x	x	x	x	x
27	rutin	350	609	301, 300			kaempferol-3-glucoside	Flavonols	x	x			
28	galloyl-bisHHDP-glucose	280	935	391, 301, 633			ellagic acid	Hydroxybenzoic acid derivatives	x				
29	cinnamic acid-3-*O*-hexoside	280, 350	355	309, 147, 207, 248			caffeic acid	Hydroxycinnamic acid derivatives	x	x	x	x	x
30	isoquercetin	350	463	301, 300	179, 151		quercetin-3-glucoside	Flavonols	x	x	x	x	x
31	kaempferol-3-coumaroylhexoside	350	593	447, 285, 257, 229			kaempferol-3-glucoside	Flavonols			x	x	x
32	quercetin-3-*O*-β-glucuronide	280, 350	477	301	179, 151		quercetin-3-glucoside	Flavonols	x	x	x	x	x
33	isorhamnetin-3-*O*-glucoside	350	477	433, 301, 179			isorhamnetin-3-glucoside	Flavonols		x	x	x	x
34	kaempferol-*O*-hexoside	350	447	284, 285			kaempferol-3-glucoside	Flavonols			x	x	x
35	caffeic acid der.	350	417	223, 179, 383	179, 135		caffeic acid	Hydroxycinnamic acid derivatives			x	x	x
36	kaempferol-3-*O*-β-glucuronide	280, 350	461	285	257, 267, 241		kaempferol-3-glucoside	Flavonols	x	x	x	x	x
37	isorhamnetin-3-*O*-β-glucuronide	350	491	315	300		isorhamnetin-3-glucoside	Flavonols	x	x	x	x	x
38	kaempferol acetyl hexoside	350	489	285	257, 267, 241		kaempferol-3-glucoside	Flavonols	x	x	x	x	x

der., derivative; [M⁻H]⁻, pseudo-molecular ion identified in negative ion mode; M⁺, pseudo-molecular ion identified in positive ion mode ion.
