# Supplementary material for: Changes in the Aroma Profile and Phenolic Compound Contents of Different Strawberry Cultivars during Ripening
Source: Plants (Basel). 2024 May 20;13(10):1419. doi: 10.3390/plants13101419 (PMC11125040; doi:10.3390/plants13101419)
Supplement: Supplementary file 1 [file plants-13-01419-s001.zip › Supplementary Material S3 Correlations.pdf]

Supplementary Material: S3 Pearson correlation among the measured parameters of strawberry fruit at different ripening stages

Table S3-1 Pearson correlation among the colour parameters, contents of phenolic compounds, enzyme activity and contents of volatile organic compounds

Pearson correlation	L*	a*	b*	C*	h°	Anthocyanins	Flavanols	Flavonols	Hydroxybenzoic acid der.	Hydroxycinnamic acid der.	Total Phenolic Compounds	POD	PPO	Total VOCs	Alcohols	Aldehydes	Esters
**Anthocyanins**	-0.82 ***	nd	-0.88 ***	nd	-0.70 ***												
**Flavanols**	nd	-0.67 **	nd	-0.73 ***	nd	nd											
**Flavonols**	nd	nd	nd	nd	nd	nd	0.64 **										
**Hydroxybenzoic acid der.**	nd	nd	nd	nd	nd	nd	nd	nd									
**Hydroxycinnamic acid der.**	-0.73 ***	nd	-0.77 ***	nd	-0.69 **	0.68 ***	nd	nd	0.57 *								
**Total Phenolic Compounds**	-0.57 **	nd	-0.66 ***	-0.52 **	nd	0.82 ***	nd	nd	nd	0.54 **							
**POD**	-0.55 *	nd	-0.56 *	nd	-0.55 *	0.57 **	nd	nd	nd	0.49 *	0.48 *						
**PPO**	nd	-0.73 ***	nd	-0.60 *	0.52 *	nd	0.75 ***	nd	nd	nd	nd	ns					
**Total VOCs**	nd	nd	nd	nd	nd	nd	nd	nd	nd	nd	nd	ns	ns				
**Alcohols**	nd	nd	nd	nd	nd	nd	nd	nd	nd	nd	nd	ns	ns	ns			
**Aldehydes**	0.54 *	-0.69 **	nd	nd	0.68 **	nd	0.58 *	0.59 *	nd	-0.65 **	nd	-0.64 **	0.76 ***	ns	0.66 ***		
**Esters**	nd	nd	nd	nd	nd	nd	nd	nd	nd	nd	nd	0.58 *	ns	ns	ns	ns	
**Terpenoids**	-0.56 *	nd	-0.70 **	-0.50 *	nd	0.72 ***	nd	nd	nd	nd	nd	ns	ns	ns	ns	ns	ns

Der., derivatives; ns, not significant; *, ** and ***: significant correlation at $p<0.05$, $p<0.01$ and at $p<0.001$, respectively.

Table S3-2 Pearson correlation among the colour parameters and contents of individual anthocyanins

**Pearson correlation**	**L***	**a***	**b***	**C***	**h°**	**cy-3-O-gal**	**cy-3-O-glc**	**plg-3-O-glc**	**plg-3-O-rut**	**plg-3-O-ara**	**cy-3-(6″malonyl)glc**	**plg der.**	**plg-3-(6″malonyl)glc**	**5-pyranoplg-3-glc**
**cy-3-O-gal**	-0.79 ***	nd	-0.82 ***	-0.45 *	-0.65 **									
**cy-3-O-glc**	-0.81 ***	-0.84 ***	-0.74 **	-0.82 ***	nd	0.92 ***								
**plg-3-O-glc**	-0.79 ***	nd	-0.86 ***	-0.44 *	-0.65 **	0.95 ***	0.98 ***							
**plg-3-O-rut**	-0.91 ***	-0.96 ***	-0.90 ***	-0.96 ***	nd	0.87 ***	0.96 ***	0.98 ***						
**plg-3-O-ara**	nd	nd	nd	nd	nd	nd	nd	nd	ns					
**cy-3-(6″malonyl)glc**	-0.70 **	0.54 *	-0.62 *	nd	-0.72 **	nd	nd	nd	ns	ns				
**plg der.**	nd	nd	nd	nd	nd	nd	nd	nd	ns	ns	0.58 *			
**plg-3-(6″malonyl)glc**	nd	nd	nd	nd	nd	nd	nd	nd	ns	ns	0.72 **	0.70 *		
**5-pyranoplg-3-glc**	nd	-1.00 *	nd	nd	nd	nd	nd	nd	1.00 *	1.00 *	ns	ns	ns	
**plg-3-O-acetylglc**	nd	nd	nd	nd	nd	nd	nd	nd	ns	ns	1.00 **	ns	-0.84 *	ns

Gal, galactoside; glc, glucoside; rut, rutinoside; ara, arabinoside; plg, pelargonidin, cy, cyanidin; der., derivative; ns, not significant. *, ** and ***: significant correlation at $p<0.05$, $p<0.01$ and at $p<0.001$, respectively.
