# Supplementary material for: Changes in the Aroma Profile and Phenolic Compound Contents of Different Strawberry Cultivars during Ripening
Source: Plants (Basel). 2024 May 20;13(10):1419. doi: 10.3390/plants13101419 (PMC11125040; doi:10.3390/plants13101419)
Supplement: Supplementary file 1 [file plants-13-01419-s001.zip › Supplementary Material S4 Aroma.pdf]

Supplementary Material: S4 Content of individual volatile organic compounds (µg g^{-1} dry weight) in the 5 studied strawberry cultivars at 5 different ripening stages

Table S4-1 Content of individual volatile organic compounds (µg g^{-1} dry weight) in the 'Aprica' strawberry cultivar at 5 different ripening stages

		Rt (min)	Sensory descriptors	'Aprica' green (R1)	white (R2)	ripe (R3)	fully ripe (R4)	overripe (R5)
**Alcohols**	1-penten-3-ol	8.58	butter, pungent	0.50 ± 0.02	0.29 ± 0.03	0.17 ± 0.02	0.18 ± 0.01	nd
	pent-(2Z)-enol	10.95	green, plastic, rubber	0.09 ± 0.00	nd	nd	nd	nd
	1-hexanol	11.31	resin, flower, green	0.26 ± 0.03	0.14 ± 0.01	0.16 ± 0.03	0.26 ± 0.03	0.36 ± 0.05
**Aldehydes**	pentanal	5.34	almond, malt, pungent	0.46 ± 0.02	nd	nd	nd	nd
	hexanal	7.14	grass, tallow, fat	12.31 ± 1.35	5.29 ± 0.25	4.74 ± 0.39	6.58 ± 0.35	5.67 ± 0.73
	pent-(2E)-enal	8.08	strawberry, fruit, tomato	0.36 ± 0.02	0.40 ± 0.04	0.15 ± 0.01	0.16 ± 0.01	nd
	cis-hex-3-enal	8.14	leaf, green	0.88 ± 0.12	0.31 ± 0.03	0.16 ± 0.04	0.18 ± 0.01	0.13 ± 0.02
	heptanal	8.97	fat, citrus, rancid	0.22 ± 0.01	0.16 ± 0.00	nd	nd	nd
	hex-(2Z)-enal	9.31	na	1.40 ± 0.05	0.85 ± 0.07	0.46 ± 0.03	0.47 ± 0.03	0.24 ± 0.02
	hex-(2E)-enal	9.61	green, leaf	48.38 ± 2.18	29.15 ± 2.27	16.95 ± 0.98	17.95 ± 1.07	8.89 ± 0.92
	octanal	10.57	fat, soap, lemon, green	nd	nd	nd	0.10 ± 0.01	0.09 ± 0.01
	2,4-(E,E)-hexadienal	11.89	green	nd	0.62 ± 0.04	0.37 ± 0.05	0.42 ± 0.03	0.27 ± 0.02
**Esters**	methyl butyrate	5.41	ether, fruit, sweet	nd	nd	13.61 ± 0.93	35.56 ± 4.00	33.12 ± 3.14
	ethyl butyrate	6.32	apple	nd	nd	0.37 ± 0.13	3.39 ± 0.99	31.70 ± 8.10
	isopropyl butyrate	6.39	pungent, fruit	nd	nd	0.20 ± 0.03	1.25 ± 0.33	1.26 ± 0.28
	ethyl isovalerate	6.86	apple	nd	nd	nd	nd	0.26 ± 0.05
	butyl acetate	6.96	pear	nd	nd	0.26 ± 0.04	0.93 ± 0.15	1.29 ± 0.20
	2-methylbutyl acetate	7.81	overripe fruit, banana, fruit, sweet	nd	nd	0.19 ± 0.03	0.31 ± 0.03	0.45 ± 0.07
	methyl hexanoate	8.98	fruit, fresh, sweet	nd	nd	18.62 ± 1.67	34.75 ± 3.24	41.38 ± 5.51
	butyl butanoate	9.48	peach, apple, berry	nd	nd	nd	nd	2.63 ± 0.72
	isopropyl hexanoate	9.68	berry, pineapple, fresh	nd	nd	nd	0.50 ± 0.09	0.63 ± 0.09
	ethyl hexanoate	9.73	apple peel, fruit	nd	nd	0.12 ± 0.03	0.75 ± 0.08	22.86 ± 5.95
	2-heptyl acetate	10.14	woody, fruity, rum-like	nd	nd	nd	nd	0.17 ± 0.02
	3-methylbutyl butanoate	10.19	fruit	nd	nd	nd	nd	0.15 ± 0.03
	hexyl acetate	10.30	fruit, herb	nd	nd	0.72 ± 0.13	1.86 ± 0.27	3.06 ± 0.41
	2-methyl hexanoate	10.60	cheesy	nd	nd	0.07 ± 0.01	0.15 ± 0.02	0.20 ± 0.03
	hex-(2E)-enyl acetate	11.08	banana peel, apple skin, privet, fresh	nd	nd	0.37 ± 0.05	0.33 ± 0.03	0.26 ± 0.02
	methyl octanoate	11.70	orange	nd	nd	0.24 ± 0.01	0.33 ± 0.06	0.47 ± 0.16
	1-methylhexyl butanoate	11.75	fruit	nd	nd	nd	0.29 ± 0.01	1.13 ± 0.09
	hexyl isobutyrate	11.97	green, fruit, apple, pear	nd	nd	nd	nd	1.26 ± 0.25
	octyl acetate	12.68	fruit	nd	nd	0.28 ± 0.05	0.45 ± 0.02	0.82 ± 0.09
**Terpenoids**	limonene	9.18	lemon, orange	0.11 ± 0.01	0.14 ± 0.01	0.09 ± 0.01	0.15 ± 0.02	0.20 ± 0.03
	linalool	13.59	flower, lavender	nd	nd	0.10 ± 0.01	0.24 ± 0.02	0.28 ± 0.02

Rt, retention time, nd, not detected, na, not available.

Table S4-2 Content of individual volatile organic compounds (µg g^{-1} dry weight) in the 'Asia' strawberry cultivar at 5 different ripening stages

		Rt (min)	Sensory descriptors	'Asia'				
				green (R1)	white (R2)	ripe (R3)	fully ripe (R4)	overripe (R5)
**Alcohols**	1-penten-3-ol	8.59	butter, pungent	0.24 ± 0.02	0.19 ± 0.01	0.12 ± 0.02	0.12 ± 0.02	0.12 ± 0.01
	1-hexanol	11.31	resin, flower, green	0.18 ± 0.02	0.19 ± 0.02	0.21 ± 0.02	0.26 ± 0.06	0.25 ± 0.02
**Aldehydes**	pentanal	5.33	almond, malt, pungent	0.31 ± 0.04	nd	nd	nd	nd
	hexanal	7.14	grass, tallow, fat	8.04 ± 0.74	6.53 ± 0.65	5.71 ± 0.65	6.56 ± 0.62	6.92 ± 1.28
	pent-(2E)-enal	8.09	strawberry, fruit, tomato	0.23 ± 0.01	0.18 ± 0.01	0.15 ± 0.02	0.16 ± 0.01	0.13 ± 0.03
	cis-hex-3-enal	8.14	leaf, green	0.45 ± 0.03	0.33 ± 0.03	0.18 ± 0.03	0.15 ± 0.01	0.19 ± 0.04
	heptanal	8.97	fat, citrus, rancid	0.15 ± 0.01	0.14 ± 0.01	nd	nd	nd
	hex-(2Z)-enal	9.31	na	1.00 ± 0.06	0.73 ± 0.05	0.46 ± 0.05	0.51 ± 0.04	0.40 ± 0.05
	hex-(2E)-enal	9.61	green, leaf	32.61 ± 1.86	24.90 ± 1.78	15.97 ± 1.85	16.61 ± 1.79	12.81 ± 1.74
	hept-(2E)-enal	11.07	fresh, fatty, pungent, almond, green	0.06 ± 0.00	nd	nd	nd	nd
	nonanal	11.79	fat, citrus, green	0.08 ± 0.01	0.11 ± 0.01	0.10 ± 0.01	0.14 ± 0.01	0.14 ± 0.01
	2,4-(E,E)-hexadienal	11.89	green	nd	0.58 ± 0.02	0.48 ± 0.06	0.55 ± 0.09	0.43 ± 0.02
	benzaldehyde	13.66	almond, burnt sugar	0.06 ± 0.01	0.11 ± 0.01	0.13 ± 0.03	0.16 ± 0.01	0.14 ± 0.03
**Esters**	methyl butyrate	5.41	ether, fruit, sweet	nd	nd	2.35 ± 0.25	11.28 ± 0.89	24.31 ± 1.74
	ethyl butyrate	6.34	apple	nd	nd	nd	0.40 ± 0.06	0.88 ± 0.23
	isopropyl butyrate	6.39	pungent, fruit	nd	nd	nd	0.87 ± 0.13	2.00 ± 0.40
	butyl acetate	6.98	pear	nd	nd	nd	0.15 ± 0.02	0.34 ± 0.05
	2-methylbutyl acetate	7.82	fruit	nd	nd	nd	0.33 ± 0.05	0.46 ± 0.05
	methyl hexanoate	8.97	fruit, fresh, sweet	nd	nd	0.59 ± 0.04	2.39 ± 0.32	5.58 ± 0.30
	dimethyl sulfite	9.07	na	nd	nd	nd	0.11 ± 0.02	nd
	butyl butanoate	9.49	fruit	nd	nd	nd	nd	0.15 ± 0.03
	ethyl hexanoate	9.73	apple peel, fruit	nd	nd	nd	0.06 ± 0.01	0.36 ± 0.04
	hexyl acetate	10.30	fruit, herb	nd	0.09 ± 0.01	0.21 ± 0.02	0.36 ± 0.07	0.47 ± 0.08
	hex-(2E)-enyl acetate	11.07	banana peel, apple skin, privet, fresh	nd	0.13 ± 0.02	0.32 ± 0.02	0.47 ± 0.13	0.54 ± 0.11
	hex-(2E)-enyl butyrate	12.68	apple, banana, fruity, orchid, grassy, green	nd	nd	nd	0.13 ± 0.03	0.15 ± 0.02
**Terpenoids**	myrcene	8.47	balsamic, must, spice	nd	nd	nd	0.14 ± 0.02	0.19 ± 0.03
	limonene	9.16	lemon, orange	nd	0.12 ± 0.02	0.08 ± 0.01	0.14 ± 0.02	0.15 ± 0.02
	linalool	13.59	flower, lavender	nd	nd	0.19 ± 0.03	0.50 ± 0.06	0.52 ± 0.03

Rt, retention time, nd, not detected, na, not available.

Table S4-3 Content of individual volatile organic compounds (μg g^{-1} dry weight) in the 'CIVN766' strawberry cultivar at 5 different ripening stages

		Rt (min)	Sensory descriptors	'CIVN766'				
				green (R1)	white (R2)	ripe (R3)	fully ripe (R4)	overripe (R5)
**Alcohols**	1-penten-3-ol	8.58	butter, pungent	0.51 ± 0.05	0.25 ± 0.02	0.15 ± 0.02	0.13 ± 0.02	0.14 ± 0.02
	pent-(2Z)-enol	10.94	green, plastic, rubber	0.08 ± 0.01	0.05 ± 0.00	nd	nd	nd
	1-hexanol	11.30	resin, flower, green	0.35 ± 0.03	0.18 ± 0.02	0.17 ± 0.03	0.24 ± 0.03	0.21 ± 0.02
	benzyl alcohol	19.66	sweet, flower	nd	nd	0.10 ± 0.00	0.17 ± 0.02	0.13 ± 0.01
**Aldehydes**	pentanal	5.33	almond, malt, pungent	0.47 ± 0.04	0.27 ± 0.03	nd	nd	nd
	hexanal	7.13	grass, tallow, fat	8.18 ± 0.53	8.03 ± 0.59	7.04 ± 0.43	8.71 ± 0.67	5.60 ± 0.21
	pent-(2E)-enal	8.07	strawberry, fruit, tomato	0.34 ± 0.03	0.22 ± 0.02	0.12 ± 0.01	nd	nd
	cis-hex-3-enal	8.13	leaf, green	0.60 ± 0.06	0.35 ± 0.01	0.19 ± 0.01	0.24 ± 0.04	0.12 ± 0.01
	heptanal	8.97	fat, citrus, rancid	0.15 ± 0.02	0.10 ± 0.02	nd	nd	nd
	hex-(2Z)-enal	9.30	na	1.07 ± 0.06	0.76 ± 0.04	0.41 ± 0.04	0.40 ± 0.04	0.35 ± 0.11
	hex-(2E)-enal	9.61	green, leaf	31.78 ± 1.68	24.97 ± 1.43	13.24 ± 1.06	10.10 ± 0.40	7.92 ± 0.48
	octanal	10.56	fat, soap, lemon, green	0.05 ± 0.01	0.05 ± 0.00	0.05 ± 0.00	0.10 ± 0.01	0.06 ± 0.01
	nonanal	11.78	fat, citrus, green	0.09 ± 0.01	0.09 ± 0.01	0.10 ± 0.01	0.17 ± 0.02	0.14 ± 0.01
	2,4-(E,E)-hexadienal	11.89	green	nd	nd	0.34 ± 0.04	0.38 ± 0.04	0.26 ± 0.02
	benzaldehyde	13.65	almond, burnt sugar	nd	nd	0.10 ± 0.00	0.17 ± 0.02	0.13 ± 0.01
**Esters**	methyl butyrate	5.41	ether, fruit, sweet	nd	nd	2.47 ± 0.21	7.05 ± 0.40	8.09 ± 0.45
	ethyl butyrate	6.34	apple	nd	nd	nd	0.60 ± 0.06	0.89 ± 0.07
	isopropyl butyrate	6.39	pungent, fruit	nd	nd	0.86 ± 0.19	1.93 ± 0.16	2.03 ± 0.10
	butyl acetate	6.96	pear	nd	nd	0.41 ± 0.05	0.98 ± 0.04	0.73 ± 0.04
	methyl hexanoate	8.97	fruit, fresh, sweet	nd	nd	0.31 ± 0.01	0.70 ± 0.05	0.80 ± 0.09
	methanethiol butyrate	9.22	cabbage, putrid, garlic, sulfury, animal, cheese	nd	nd	nd	0.06 ± 0.01	0.07 ± 0.01
	butyl butanoate	9.49	fruit	nd	nd	0.21 ± 0.04	0.83 ± 0.04	1.17 ± 0.07
	ethyl hexanoate	9.73	apple peel, fruit	nd	nd	nd	nd	0.03 ± 0.01
	3-methylbutyl butanoate	9.95	fruit	nd	nd	nd	0.13 ± 0.01	0.14 ± 0.01
	3-methylbutyl 2-methyl propanoate	10.20	fruity, waxy, apricot, pineapple, green	nd	nd	nd	0.14 ± 0.01	0.15 ± 0.01
	hexyl acetate	10.30	fruit, herb	0.14 ± 0.02	0.20 ± 0.04	0.48 ± 0.04	0.78 ± 0.04	0.75 ± 0.06
	hex-(3E)-enyl acetate	10.90	fruity	nd	0.05 ± 0.00	0.07 ± 0.01	0.10 ± 0.01	0.07 ± 0.01
	hex-(2E)-enyl acetate	11.08	banana peel, apple skin, privet, fresh	nd	0.25 ± 0.05	0.48 ± 0.07	0.67 ± 0.11	0.53 ± 0.08
	hex-(2E)-enyl butyrate	12.69	apple, banana, fruity, orchid, grassy, green	nd	nd	nd	0.26 ± 0.03	0.24 ± 0.02
	octyl butyrate	14.81	fruit	nd	nd	nd	0.13 ± 0.01	0.14 ± 0.01
**Furanones**	mesifuran	14.68	caramel, sweet, mildew	nd	nd	nd	0.15 ± 0.02	0.15 ± 0.02
**Terpenoids**	limonene	9.16	lemon, orange	nd	nd	0.05 ± 0.01	0.07 ± 0.01	0.05 ± 0.00
	linalool	13.59	flower, lavender	nd	nd	0.07 ± 0.01	0.12 ± 0.01	0.10 ± 0.01

Rt, retention time, nd, not detected, na, not available.

Table S4-4 Content of individual volatile organic compounds ($\mu g\ g^{-1}$ dry weight) in the 'Clery' strawberry cultivar at 5 different ripening stages

		Rt (min)	Sensory descriptors	'Clery'				
				green (R1)	white (R2)	ripe (R3)	fully ripe (R4)	overripe (R5)
**Alcohols**	1-penten-3-ol	8.57	butter, pungent	0.85 ± 0.03	0.95 ± 0.13	0.48 ± 0.02	0.36 ± 0.03	nd
	pent-(2Z)-enol	10.90	green, plastic, rubber	0.07 ± 0.00	0.07 ± 0.01	nd	nd	nd
	hex-(3Z)-enol	10.94	grass	nd	nd	0.09 ± 0.01	nd	nd
	1-hexanol	11.29	resin, flower, green	0.40 ± 0.03	0.43 ± 0.09	0.31 ± 0.04	0.25 ± 0.03	0.40 ± 0.06
	2-hexen-1-ol	11.88	green, leaf, walnut	nd	nd	nd	nd	0.39 ± 0.06
**Aldehydes**	pentanal	5.35	almond, malt, pungent	0.39 ± 0.02	0.30 ± 0.04	nd	nd	nd
	hexanal	7.11	grass, tallow, fat	9.85 ± 0.30	9.36 ± 0.56	7.13 ± 0.52	6.83 ± 0.80	8.05 ± 0.75
	pent-(2E)-enal	8.08	strawberry, fruit, tomato	0.26 ± 0.01	0.30 ± 0.02	0.15 ± 0.01	0.18 ± 0.01	0.14 ± 0.02
	cis-hex-3-enal	8.11	leaf, green	0.36 ± 0.02	0.41 ± 0.05	0.16 ± 0.01	0.15 ± 0.01	0.16 ± 0.01
	heptanal	8.99	fat, citrus, rancid	0.10 ± 0.01	nd	nd	nd	nd
	hex-(2Z)-enal	9.28	na	0.94 ± 0.02	0.99 ± 0.12	0.43 ± 0.02	0.38 ± 0.03	0.32 ± 0.04
	hex-(2E)-enal	9.59	green, leaf	30.58 ± 0.70	28.07 ± 1.76	14.14 ± 0.49	12.15 ± 0.78	10.52 ± 1.17
	octanal	10.56	fat, soap, lemon, green	nd	nd	0.05 ± 0.01	0.07 ± 0.00	0.08 ± 0.02
	nonanal	11.79	fat, citrus, green	0.06 ± 0.01	0.08 ± 0.01	0.09 ± 0.01	nd	nd
	2,4-(E,E)-hexadienal	11.90	green	0.85 ± 0.03	0.95 ± 0.13	0.48 ± 0.02	0.36 ± 0.03	nd
	benzaldehyde	13.63	almond, burnt sugar	0.04 ± 0.00	0.09 ± 0.01	0.08 ± 0.01	0.12 ± 0.01	0.13 ± 0.02
**Esters**	methyl butyrate	5.40	ether, fruit, sweet	nd	nd	22.64 ± 1.82	30.22 ± 2.29	25.35 ± 2.18
	ethyl butyrate	6.32	apple	nd	nd	3.20 ± 1.23	2.23 ± 0.27	4.22 ± 0.83
	isopropyl butyrate	6.36	pungent, fruit	nd	nd	nd	2.24 ± 0.40	2.41 ± 0.14
	butyl acetate	6.94	pear	nd	nd	0.68 ± 0.16	0.69 ± 0.08	1.14 ± 0.07
	methyl 4-methyl pentanoate	8.22	na	nd	nd	0.13 ± 0.01	0.16 ± 0.01	0.14 ± 0.01
	methyl hexanoate	8.96	fruit, fresh, sweet	nd	0.23 ± 0.04	11.21 ± 0.54	10.59 ± 1.13	9.71 ± 0.82
	methanethiol butyrate	9.22	cabbage, putrid, garlic, sulfury, animal, cheese	nd	nd	nd	0.07 ± 0.01	0.10 ± 0.01
	butyl butanoate	9.47	fruit	nd	nd	1.49 ± 0.65	1.88 ± 0.66	2.89 ± 0.45
	isopropyl hexanoate	9.67	berry, pineapple, fresh, logenberry, fruity	nd	nd	nd	0.57 ± 0.11	0.55 ± 0.06
	ethyl hexanoate	9.72	apple peel, fruit	nd	nd	0.23 ± 0.08	0.55 ± 0.07	1.11 ± 0.40
	3-methylbutyl butanoate	9.94	fruit	nd	nd	nd	0.15 ± 0.03	0.24 ± 0.02
	hexyl acetate	10.29	fruit, herb	0.08 ± 0.00	0.17 ± 0.01	0.92 ± 0.10	0.93 ± 0.13	1.46 ± 0.10
	2-methyl hexanoate	10.62	cheesy	nd	nd	0.08 ± 0.01	0.07 ± 0.01	0.10 ± 0.02
	hex-(2E)-enyl acetate	11.07	banana peel, apple skin, privet, fresh	0.10 ± 0.01	0.24 ± 0.01	0.59 ± 0.09	0.28 ± 0.02	0.55 ± 0.13
	methyl octanoate	11.69	orange	nd	nd	0.29 ± 0.02	0.30 ± 0.01	0.25 ± 0.02
	1-methylhexyl butanoate	11.75	fruit	nd	nd	nd	nd	0.61 ± 0.11
	hexyl butyrate	11.97	apple peel	nd	nd	nd	nd	1.41 ± 0.13
	octyl acetate	12.69	fruit	nd	nd	0.38 ± 0.05	0.43 ± 0.05	0.54 ± 0.05
	hexyl hexanoate	14.67	apple peel, peach	nd	nd	nd	nd	0.12 ± 0.02
	octyl butyrate	14.79	fruit	nd	nd	0.22 ± 0.08	0.40 ± 0.06	0.64 ± 0.08
**Terpenoids**	limonene	9.14	lemon, orange	nd	nd	0.09 ± 0.01	0.09 ± 0.02	0.09 ± 0.02
	linalool	13.58	flower, lavender	nd	nd	0.15 ± 0.02	0.19 ± 0.02	0.20 ± 0.03

Rt, retention time, nd, not detected, na, not available.

Table S4-5 Content of individual volatile organic compounds (μg g^{-1} dry weight) in the 'Malwina' strawberry cultivar at 5 different ripening stages

		Rt (min)	Sensory descriptors	'Malwina'				
				green (R1)	white (R2)	ripe (R3)	fully ripe (R4)	overripe (R5)
**Alcohols**	1-penten-3-ol	8.58	butter, pungent	0.60 ± 0.03	0.32 ± 0.04	0.22 ± 0.02	0.23 ± 0.01	0.18 ± 0.02
	pent-(2Z)-enol	10.94	green, plastic, rubber	0.09 ± 0.01	0.05 ± 0.01	0.05 ± 0.00	nd	nd
	1-hexanol	11.30	resin, flower, green	0.43 ± 0.04	0.20 ± 0.02	0.18 ± 0.01	0.22 ± 0.01	0.21 ± 0.02
**Aldehydes**	pentanal	5.33	almond, malt, pungent	0.55 ± 0.04	0.37 ± 0.05	nd	nd	nd
	hexanal	7.13	grass, tallow, fat	15.52 ± 1.14	11.11 ± 0.60	9.92 ± 0.35	11.44 ± 0.81	9.68 ± 0.42
	pent-(2E)-enal	8.07	strawberry, fruit, tomato	0.39 ± 0.02	0.26 ± 0.02	0.18 ± 0.03	0.16 ± 0.01	0.16 ± 0.01
	cis-hex-3-enal	8.13	leaf, green	0.73 ± 0.03	0.42 ± 0.04	0.21 ± 0.02	0.22 ± 0.01	0.16 ± 0.01
	heptanal	8.97	fat, citrus, rancid	0.13 ± 0.01	0.10 ± 0.02	nd	nd	nd
	hex-(2Z)-enal	9.30	na	1.66 ± 0.06	1.03 ± 0.08	0.59 ± 0.05	0.62 ± 0.03	0.45 ± 0.03
	hex-(2E)-enal	9.61	green, leaf	52.91 ± 1.73	33.57 ± 2.79	20.09 ± 1.86	20.76 ± 1.08	15.57 ± 0.88
	octanal	10.56	fat, soap, lemon, green	nd	nd	0.06 ± 0.01	0.06 ± 0.00	0.08 ± 0.01
	hept-(2E)-enal	11.07	fresh, fatty, pungent, almond, green	0.12 ± 0.01	0.11 ± 0.01	0.23 ± 0.03	nd	nd
	nonanal	11.78	fat, citrus, green	0.09 ± 0.01	0.08 ± 0.02	0.09 ± 0.01	0.11 ± 0.01	0.11 ± 0.01
	2,4-(E,E)-hexadienal	11.89	green	1.08 ± 0.04	0.66 ± 0.06	0.41 ± 0.03	0.44 ± 0.02	0.34 ± 0.01
	benzaldehyde	13.65	almond, burnt sugar	nd	nd	0.08 ± 0.01	0.08 ± 0.01	0.09 ± 0.00
**Esters**	methyl butyrate	5.41	ether, fruit, sweet	nd	nd	2.90 ± 0.85	5.70 ± 0.38	7.60 ± 0.21
	ethyl butyrate	6.34	apple	nd	nd	1.08 ± 0.10	2.79 ± 0.61	8.89 ± 0.34
	isopropyl butyrate	6.39	pungent, fruit	nd	nd	0.25 ± 0.01	0.59 ± 0.12	1.92 ± 0.04
	ethyl isovalerate	6.88	apple	nd	nd	nd	nd	0.25 ± 0.00
	butyl acetate	6.96	pear	nd	nd	0.35 ± 0.06	0.73 ± 0.04	0.89 ± 0.02
	2-methylbutyl acetate	7.80	fruit	nd	nd	0.53 ± 0.03	0.81 ± 0.09	0.86 ± 0.03
	methyl 4-methyl pentanoate	8.22	na	nd	nd			0.11 ± 0.01
	methyl hexanoate	8.97	fruit, fresh, sweet	nd	nd	1.48 ± 0.52	2.69 ± 0.29	5.02 ± 0.12
	butyl butanoate	9.49	fruit	nd	nd	nd	nd	0.04 ± 0.01
	ethyl hexanoate	9.73	apple peel, fruit	nd		0.12 ± 0.03	0.42 ± 0.11	2.30 ± 0.45
	hexyl acetate	10.30	fruit, herb	nd	0.09 ± 0.01	0.36 ± 0.07	0.61 ± 0.04	0.77 ± 0.06
	hex-(2E)-enyl acetate	11.08	banana peel, apple skin, privet, fresh	nd	nd	nd	0.36 ± 0.03	0.35 ± 0.04
	methyl octanoate	11.71	orange	nd	nd	nd	nd	0.23 ± 0.01
	octyl acetate	12.69	fruit	nd	nd	nd	nd	0.22 ± 0.02
	2-methyloctyl butyrate	15.00	na	nd	nd	nd	nd	0.09 ± 0.01
**Terpenoids**	myrcene	8.46	balsamic, must, spice	nd	nd	nd	nd	0.12 ± 0.01
	limonene	9.16	lemon, orange	0.09 ± 0.01	0.10 ± 0.02	0.12 ± 0.01	0.12 ± 0.01	0.16 ± 0.01
	linalool	13.59	flower, lavender	nd	nd	0.23 ± 0.03	0.39 ± 0.05	0.44 ± 0.06

Rt, retention time, nd, not detected, na, not available.

Table S4-6 Coding of individual volatile organic compounds used for partial least-square discriminant analysis

Code	Group	Compound
AC1	alcohol	1-penten-3-ol
AC2	alcohol	1-hexanol
AC3	alcohol	pent-(2Z)-enol
AC4	alcohol	hex-(3Z)-enol
AC5	alcohol	2-hexen-1-ol
AC6	alcohol	benzyl alcohol
AD1	aldehyde	pentanal
AD2	aldehyde	hexanal
AD3	aldehyde	pent-(2E)-enal
AD4	aldehyde	cis-hex-3-enal
AD5	aldehyde	heptanal
AD6	aldehyde	hex-(2Z)-enal
AD7	aldehyde	hex-(2E)-enal
AD8	aldehyde	hept-(2E)-enal
AD9	aldehyde	nonanal
AD10	aldehyde	2,4-(E,E)-hexadienal
AD11	aldehyde	benzaldehyde
AD13	aldehyde	octanal
E1	ester	methyl butyrate
E2	ester	ethyl butyrate
E3	ester	isopropyl butyrate
E4	ester	butyl acetate
E5	ester	2-methylbutyl acetate
E6	ester	methyl hexanoate
E7	ester	dimethyl sulfite
E8	ester	butyl butanoate
E9	ester	ethyl hexanoate
E10	ester	hexyl acetate
E11	ester	hex-(2E)-enyl acetate
E12	ester	hex-(2E)-enyl butyrate
E13	ester	butyl acetate
E14	ester	methyl 4-methyl pentanoate
E15	ester	methanethiol butyrate
E16	ester	isopropyl hexanoate
E17	ester	3-methylbutyl butanoate
E18	ester	2-methyl hexanoate
E19	ester	methyl octanoate
E20	ester	1-methylhexyl butanoate
E21	ester	hexyl butyrate
E22	ester	octyl acetate
E23	ester	hexyl hexanoate
E24	ester	octyl butyrate
E25	ester	ethyl isovalerate
E26	ester	2-heptyl acetate
E27	ester	hexyl isobutyrate
E28	ester	3-methylbutyl 2-methyl propanoate
E29	ester	hex-(3E)-enyl acetate
E30	ester	2-methyloctyl butyrate
F1	furan	mesifuran
T1	terpene	myrcene
T2	terpene	limonene
T3	terpene	linalool
